# Evaluation des pratiques d’antibioprophylaxie chirurgicale dans un Hopital Universitaire du Centre Tunisien

**DOI:** 10.11604/pamj.2018.30.191.14861

**Published:** 2018-07-02

**Authors:** Hayett Harbi, Latifa Merzougui, Mohamed Hafedh Barhoumi, Hedi Rebai, Sofiene Abdelkefi, Rafik El Kamel, Tarek Barhoumi

**Affiliations:** 1 Direction Régionale de la Santé, Kairouan, Tunisie; 2 Service d’Hygiène Hospitalière CHU Ibn El Jazzar, Kairouan, Tunisie; 3 Service d’Anesthésie et de Réanimation CHU Ibn El Jazzar, Kairouan, Tunisie; 4 Service d’Orthopédie CHU Ibn El Jazzar, Kairouan, Tunisie; 5 Service de Chirurgie Générale CHU Ibn El Jazzar Kairouan, Tunisie; 6 Service d’Urologie CHU Ibn El Jazzar, Kairouan, Tunisie

**Keywords:** Evaluation, pratiques professionnelles, antibioprophylaxie, audit clinique, amélioration, Evaluation, professional practices, antibiotic prophylaxis, clinical audit, improvement

## Abstract

L'antibioprophylaxie (ATBP) est l'une des mesures spécifiques de prévention des infections du site opératoire dont l'impact a été quantifié dans les interventions chirurgicales propres et propres contaminées. L'objectif de notre étude est d'évaluer la conformité des pratiques d'ATBP et le respect des prescripteurs des protocoles adoptés au sein de notre établissement. Notre travail est une étude observationnelle rétrospective, de type « Audit Clinique », évaluant les pratiques d'ATBP dans notre établissement durant le mois de mars 2015. Le critère de jugement principal était la conformité globale des pratiques observées aux 5 critères majeurs définis par la haute autorité française de santé (HAS). Nous avons adopté le référentiel de la société française d'anesthésie réanimation parue en 2010. Nous avons inclus 150 patients opérés des services de chirurgie générale, d'orthopédie et d'urologie. Le taux de conformité globale était de 33,3%. La conformité de chacun des 5 critères majeurs définis par la HAS était de 74% pour l'indication; 84% pour le délai injection-incision; 60% pour le choix de l'ATB; 89,3% pour la dose de 1^ère^ injection et 72% pour la durée de l'ATBP. La conformité était variable selon le service, meilleure en urologie ; en cas de chirurgie programmée et quand le prescripteur est un réanimateur. Une stratégie globale intégrant organisation, éducation et restriction, pourrait permettre une réelle amélioration des taux de conformité des pratiques d'ATBP. La réalisation d'audits successifs devrait s'inscrire dans une démarche régulière, afin d'évaluer l'impact des actions entreprises.

## Abstract (en)

Antibiotic prophylaxis (ATBP) is one of the specific measures for the prevention of surgical site infections, whose impact has been quantified in clean or clean-contaminated surgery. Our study aims to evaluate the conformity of ATBP practices and the adherence to the prescribing protocols adopted in our Hospital. We conducted a clinical audit retrospective observational study, evaluating antibiotic prophylaxis practices in our Hospital in the month of March 2015. The primary study endpoint was the overall compliance of the observed practices with the 5 major criteria defined by the French National Authority for Health (FNAH). We followed the guidelines of the French Society of Anesthesia and Intensive Care published in 2010. The study included 150 patients who had undergone surgery in the Department of General Surgery, Orthopaedics and Urology. The overall compliance rate was 33.3%. The compliance with each of the 5 major criteria defined by the FNAH was 74% for the indication; 84% for the time between injection and incision; 60% for the choice of ATB; 89.3% for the dose of the first injection and 72% for the duration of ATBP. The compliance was variable depending on the Department; better compliance was reported in the Department of Urology, in scheduled surgery and when the prescriber was an anesthetist-resuscitator. A global strategy including organization, education and restriction, could lead to a real improvement in the rate of compliance with ATBP practices. Successive audits should be carried out regularly in order to evaluate the impact of the undertaken actions.

## Introduction

L'antibioprophylaxie (ATBP) est l'une des mesures spécifiques de prévention des infections du site opératoire (ISO) dont l'impact a été quantifié dans les interventions chirurgicales propres et propres contaminées [1,2]. Elle correspond à l'administration d'un antibiotique (ATB) avant une intervention chirurgicale chez un patient non contaminé. L'objectif essentiel de cette ATBP est la réduction de l'incidence des infections postopératoires, qui représentent plus de 30% des infections nosocomiales (IN) [3,4]. L'utilisation incorrecte des ATB reste fréquente dans cette indication d'où l'intérêt de faire une évaluation des pratiques de l'ATBP. Le premier pas vers une optimisation des prescriptions est d'évaluer les pratiques de l'ATBP chirurgicale, afin de déterminer si l'utilisation préventive des ATB au bloc opératoire est conforme aux recommandations [5]. L'objectif de notre étude est d'évaluer la conformité des pratiques d'ATBP et le respect des prescripteurs des protocoles adoptés au sein de l'établissement. Le but ultime est de proposer des mesures d'intervention ciblées visant à optimiser la réalisation de l'ATBP en chirurgie afin de contribuer aux efforts de prévention des IN.

## Méthodes

Il s'agit d'une étude observationnelle rétrospective, de type « Audit Clinique », évaluant les pratiques d'ATBP dans notre établissement.

**Critères d'inclusion**: Patients opérés dans les services ayant accepté de participer à notre étude (chirurgie générale, orthopédie et urologie). Nous avons jugé une taille d'échantillon de 50 patients par service (selon «Agence Nationale d'accréditation et d'évaluation en santé (ANAES). L'Audit Clinique. Bases Méthodologiques de L'Evaluation des Pratiques Professionnelles. Avril 1999 »). Nous avons inclus les 50 premiers patients qui ont été opérés durant la période d'étude sans tenir compte de leur âge, leur sexe ou du caractère de chirurgie appliquée (programmée ou en urgence).

**Critères d'exclusion**: Patients sous antibiothérapie en préopératoire. Dossiers comportant des données manquantes (heure d'incision, heure d'injection d'ATB).

**Période d'étude**: L'étude a été menée durant un mois: mars 2015. Elle a été précédée par un pré-test pour évaluer et valider la grille d'audit avec le groupe du projet. Trois enquêteurs ont été formés par le groupe de pilotage.

**Choix des critères** : Nous avons construit notre grille en s'inspirant de celle établie par la haute autorité de santé française (HAS) dans son guide d'évaluation de l'ATBP en chirurgie propre [5] et aussi en se basant sur la revue de la littérature, en particulier: les recommandations de la société française d'anesthésie réanimation SFAR [6]. Guide méthodologique: l'audit en hygiène hospitalière: du concept à la réalisation (C.CLIN Paris-Nord 1999) [7]. Guide méthodologique: l'audit clinique: bases méthodologiques de l'évaluation des pratiques professionnelles (ANAES avril 1999) [8].

**Choix de la méthode de mesure et recueil des données**: Le critère de jugement principal était la conformité globale des pratiques observées aux 5 critères majeurs définis par la HAS: 1) ATBP indiquée et réalisée. 2) Choix de la molécule d'ATB conforme aux recommandations. 3) Horaire de la première administration adapté par rapport au moment de l'incision. 4) Conformité des posologies. 5) Durée de l'ATBP conforme aux recommandations.

**Analyse des résultats**: Les données ont été saisies et analysées au moyen d'un logiciel SPSS 20. L'analyse des résultats est basée sur le calcul d'un score « taux de conformité ». Les taux de conformité sont calculés pour chaque critère et pour chaque service. La conformité globale de la prescription est calculée pour chaque acte d'ATBP.

## Résultats

**Population d'étude**: Notre étude a inclus 150 patients opérés dans les services de chirurgie générale, orthopédie et urologie.La moyenne d'âge était de 55,26 ± 21,71 ans avec un minimum de 5 ans et un maximum de 94 ans. Le sexe ratio est de 1,94 H/F avec une prédominance masculine. Un seul patient était allergique aux β-lactamines. Parmi les 150 patients, 96% avaient un score ASA 1 ou 2.

**Caractéristiques des actes opératoires**: Parmi les 150 actes chirurgicaux de notre étude, 97 correspondaient à une classe de contamination propre (Altemeier 1) et 53 à une classe de contamination propre contaminée (Altemeier 2). Par ailleurs, 101 interventions étaient programmées, alors que 49 étaient urgentes. Parmi les 150 actes d'ATBP, 95 ont été prescrites par des chirurgiens alors que seulement 46 ont été prescrites par des anesthésistes réanimateurs (Tableau 1).

**Tableau 1:** Caractéristiques de l’acte chirurgical

Caractéristiques	N (%)	
Classe de contamination	Altemeier 1	97 (64,7)
	Altemeier 2	53 (35,3)
Type de chirurgie	Programmée	101 (67,3)
	Urgente	49 (32,7)

**Conformité de l'indication de l'ATBP**: Parmi les 150 cas étudiés, l'indication de l'ATBP était: conforme aux recommandations de la SFAR dans 111 cas (74%) : « indiquée et faite ». non conforme aux recommandations dans 39 cas (26%): « indiquée et non faite » dans 6 cas; « non indiquée et faite » dans 33 cas.

**Figure 1:**
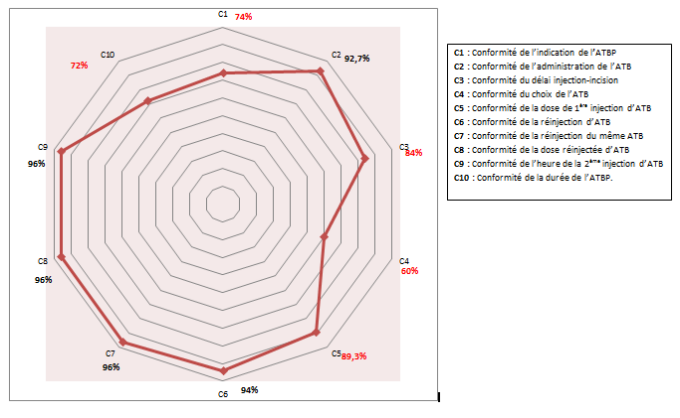
Taux de conformité globale des critères de l’antibioprophylaxie CHU Ibn El Jazzar, Kairouan, Tunisie 2015

**Conformité des autres critères**: Le critère le moins adapté était le choix de la molécule d'ATB avec un taux de conformité de 60%. La conformité du choix de l'ATB varie bien selon le service: plus importante en orthopédie (82%) qu'en chirurgie générale (36%). Ce taux varie aussi selon le type de chirurgie: conforme dans 51,5% des cas quand l'acte est pratiqué à froid et dans seulement 22,5% des cas quand l'acte est pratiqué en urgence. De même, le choix de l'ATB varie selon le prescripteur: il est de 72% quand le prescripteur est un anesthésiste réanimateur et 56% quand il s'agit d'un chirurgien. Le délai d'injection de l'ATB par rapport à l'incision a été respecté dans 84% des cas. La conformité varie selon le service de soins: plus importante en orthopédie (96%) qu'en chirurgie générale (74%) et selon le type de chirurgie: beaucoup plus importante à froid (82%) qu'en urgence (12%). La posologie de la 1^ère^ injection d'ATB en préopératoire était conforme dans 89,3% des cas. En cas de prescription d'ATBP par un chirurgien, la conformité de la dose de 1^ère^ injection était de 89%. Par contre, quand le prescripteur est un anesthésiste réanimateur, ce taux s'élève jusqu'à 92,5%. En orthopédie, letaux de conformité de la dose de 1^ère^ injection était de 96%, alors qu'en urologie ce taux était de 80%. La durée de l'ATBP a été respectée dans 72% des cas. En effet, dans 71,3% des cas, la durée de l'ATBP ne dépassait pas les 24 heures et dans un seul cas, elle était limitée à 48 heures uniquement. Par contre, dans 24% des cas elle dépassait les 48 heures (Figure 1). Le taux de conformité de ce critère varie selon le service, il était de 98% en chirurgie générale et de 90% en urologie, mais très faible en orthopédie (28%). Il est aussi plus important quand l'acte est pratiqué à froid (83%) qu'en urgence (49%).

**Figure 2:**
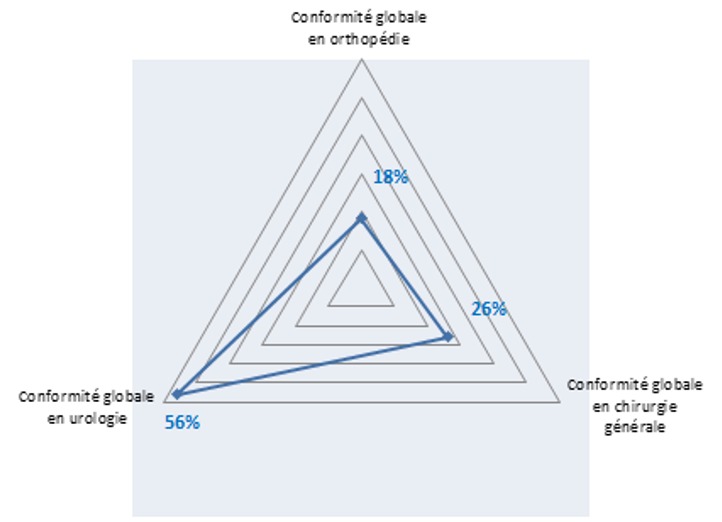
Conformité globale de l’antibioprophylaxie par service CHU Ibn El Jazzar, Kairouan, Tunisie 2015

**Conformité globale de l'ATBP**: Notre étude a inclus 150 patients, parmi lesquels 117 ATBP ont été recommandées (78%). Parmi ces dernières, 111 ATBP ont été faites (74%), parmi lesquelles 50 ont été faites correctement selon les recommandations de la SFAR (33,3%). L'analyse de la conformité globale d'ATBP a montré qu'en chirurgie générale, 13 cas d'ATBP étudiés ont été conformes (26%); en urologie, 28 cas parmi les 50 étudiés ont été conformes (56%) et en orthopédie, 9 cas d'ATBP seulement ont été conformes (18%) (Figure 2). Dans notre étude, 49 actes parmi 150 ont été pratiqués en urgence. Parmi ces 49 actes, 12 étaient conformes aux recommandations ce qui correspond à une fréquence de 25%. Dans les 101 actes programmés et étudiés, 38 étaient conformes aux recommandations ce qui correspond à un taux de conformité de 38%. Dans le cas où le prescripteur est un chirurgien, 19 prescriptions parmi 95 étaient conformes (20%). Par contre, quand le prescripteur était un médecin anesthésiste réanimateur, 28 prescriptions d'ATBP parmi 46 étaient conformes (61%) (Tableau 2).

**Tableau 2:** Conformité Globale de l’antibioprophylaxieCHU Ibn El Jazzar Kairouan Tunisie 2015

		Taux de conformité globale (%)
**Service**	Orthopédie	18
	Chirurgie Générale	26
	Urologie	56
**Prescripteur**	Chirurgien	20
	Anesthésiste Réanimateur	61
**Type de chirurgie**	Programmée	38
	En urgence	25

## Discussion

L'infection du site opératoire représente la première complication de la chirurgie et une proportion considérable des infections nosocomiales [9]. Intégrée dans le respect des mesures élémentaires d'hygiène et les bonnes pratiques chirurgicales, l'ATBP est un des outils essentiels de la maitrise du risque infectieux [10]. Ses modalités font l'objet de recommandations validées, mais sa réalisation reste imparfaite [11]. L'objectif de notre étude était d'évaluer la conformité des pratiques d'ATBP et l'adhérence des prescripteurs aux protocoles adoptés au sein de notre établissement. En l'absence de protocole local, le référentiel était la conférence de consensus de la Société Française d'Anesthésie Réanimation (SFAR) parue en 2010 (actualisation en 2011) [6]. Le but ultime est de proposer des mesures d'intervention ciblées visant à optimiser la réalisation de l'ATBP en chirurgie afin de contribuer aux efforts de prévention des IN.

**Discussion de la méthodologie**: Notre travail est un « Audit rétrospectif ». Ce type d'étude présente des points forts et aussi des vulnérabilités. L'un des avantages des études rétrospectives est d'éviter les biais souvent rencontrés dans les études prospectives tels que: le biais d'information ou d'observation et l'effet « Hawthorne ». En effet, le biais d'observation résulte d'erreurs systématiques dans la récolte de l'information. L'effet « Hawthorne » est un autre type de biais relié au comportement des participants. Par ailleurs, lors des études rétrospectives, on va éliminer des dossiers souvent pour manque de données ou d'informations. Il s'agit bien d'un biais de sélection qui peut induire un échantillon non représentatif de la population étudiée. Plusieurs moyens ont été utilisés pour prévenir ces différents biais: assurer la formation des enquêteurs. Ne pas dévoiler aux participants l'hypothèse de l'étude.

**Discussion des résultats**: Notre étude a confirmé l'écart entre les recommandations émises par les sociétés savantes et les pratiques observées. En effet, notre taux de conformité globale a été de 33,3%. Le taux de conformité globale retrouvé dans notre étude est faible en comparaison avec la littérature. En effet, les auteurs décrivent une conformité de la prophylaxie aux référentiels locaux dans plus de 80% [12]. Alerany et al ont décrit une conformité de l'ATBP au protocole local de 94,9% dans un hôpital universitaire à Barcelone [10]. Les études en Tunisie ont décrit des taux de conformité aux référentiels respectivement de 23,5% dans l'étude de Naija et al au CHU Sahloul de Sousse [13] et de 47% dans l'étude de Kallel et al au CHU Habib Bourguiba de Sfax [14]. Pour cette dernière, les auteurs ne prennent pas en considération l'intervalle de temps entre l'injection d'ATB et l'incision malgré que ce critère ait constitué un critère majeur de conformité de l'ATBP selon l'ANAES [5] et un axe important pour l'amélioration des pratiques d'ATB dans différentes études [15]. Dans notre travail, les critères les moins conformes étaient respectivement le choix de l'ATB avec un TC de 60% et la durée de l'ATBP avec un TC de 72%.Nos résultats sont très proches de ceux retrouvés dans la littérature. En effet, dans l'étude de Bull et al en Australie ce taux était de 53,3% [16]. Il était respectivement de 62% dans l'étude de Rafati et al en Iran [17], 62,4% dans l'étude de Kallel et al au CHU Habib Bourguiba de Sfax [14] et 69,6% dans l'étude de Thouverez et al en France [18]. D'autres études ont pu trouver des taux de conformité du choix de l'ATB meilleurs. Viprey et al, dans une étude menée dans un centre hospitalier à Vienne, ont trouvé un taux de conformité de 92,5% [19]. De même aux USA, Russel et al, ont retrouvé dans leur étude un taux de conformité de 97% pour le choix de l'ATB [20]. Les principales non-conformités sont représentées par des prescriptions d'ATB à spectre plus large que ceux recommandés dans les référentiels entraînant un surcoût financier considérable ainsi qu'un risque d'émergence de souches bactériennes résistantes aux ATB, non négligeable.

Dans notre étude, la durée de l'ATBP a été respectée dans 72% des cas. En effet, dans 71,3% des cas, la durée de l'ATBP ne dépassait pas les 24 heures et dans un seul cas, elle était limitée à 48 heures uniquement. Par contre, dans 24% des cas la durée de l'ATBP dépassait les 48 heures. Dans la littérature, le taux faible de conformité de la durée d'ATBP a été décrit dans l'étude de Hosoglu et al en Turquie qui ont trouvé un taux de conformité de 19,6% et que 80,4% des prescriptions s'allongeaient plus que 5 jours [21]. De même, dans une étude prospective en Palestine, la durée d'ATBP a été respectée dans uniquement 31,8% des cas [22]. Une autre étude faite par Miliani et al en France a décrit une durée d'ATBP appropriée dans 35% des cas, très allongée dans 45,2% des cas et insuffisante dans 19,8% des cas [23]. Par contre, d'autres études ont décrit des taux de conformité de ce critère plus importants: il était de 78,5% dans l'étude d'Arques et al [24] et de 100% dans l'étude de Lemtiri-Florek et al [25]. Le taux faible de la conformité de la durée de l'ATBP observé en orthopédie (TC=28%) peut être expliqué dans notre cas par la mise en place de prothèses et la crainte des chirurgiens quant au développement des ISO. De même, le taux de conformité bas observé avec les actes urgents est expliqué par la précipitation des chirurgiens à prescrire les ATB en excès, d'une façon prolongée et en utilisant des molécules à spectre large. En se basant sur la littérature, une antibioprophylaxie supérieure à 24 heures n'a aucun intérêt prouvé [26]. L'utilisation prolongée des céphalosporines de 3^ème^ génération est souvent associée à l'émergence des nouvelles souches de staphylocoques résistantes à la Méticilline (SARM) [27]. En Italie, les guides nationaux recommandent l'utilisation d'une dose unique en ATBP [28]. L'extension de l'ATBP dans les 24 heures suivant l'intervention chirurgicale est recommandée dans certains actes sévères (chirurgie générale, urologie, chirurgie cardiovasculaire, neurochirurgie) sans considération du risque de contamination [29].

**Cause des non-conformités**: Une étude des causes des non-conformités réalisée par le comité de pilotage et les différents intervenants, a mis en évidence l'existence de plusieurs facteurs limitant l'adhésion des médecins aux protocoles adoptés: un manque d'organisation au bloc opératoire ainsi qu'une mauvaise coordination entre les différents acteurs lors de la prise en charge du patient (anesthésiste, chirurgien, infirmiers et aides). Une attitude excessive des chirurgiens lors de certains actes (mise en place de prothèses) montrant leur crainte quant au développement des ISO. Le manque de formation et d'informations des intervenants des protocoles d'ATBP et des éventuelles mises à jour. Ces mêmes facteurs ont été soulignés par Cabana et al [30]. Ces derniers distinguent plusieurs types de barrières en fonction de leur influence sur les connaissances, l'attitude et le comportement des médecins. L'ATBP chirurgicale a fait preuve de son efficacité dans la réduction des ISO. Il est important de respecter les grands principes afin de limiter son impact sur l'écologie bactérienne.

**Plan d'actions d'amélioration**: un plan d'action d'amélioration des pratiques d'ATBP a été mis en place et portant essentiellement sur: **Des mesures organisationnelles**: élaboration d'un protocole local, adoption des check-lists et responsabilisation des différents acteurs. **Des mesures éducationnelles**: formation et information des acteurs de la chirurgie sur l'ATBP, ses modalités et son importance ainsi que leur sensibilisation quant au coût économique engendré par les mauvaises prescriptions d'ATB et l'émergence des nouvelles souches résistantes suite à l'utilisation massive des ATB surtout à large spectre. **Des mesures restrictives telles que**: les ordonnances pré-imprimées, la dispensation journalière individuelle nominative et l'arrêt systématique de l'ATBP après 24 à 48 heures au maximum.

## Conclusion

Notre étude a montré qu'il existe un écart important entre les pratiques observées et les recommandations des sociétés savantes. La réalisation de ce type d'audit devrait s'inscrire dans une démarche régulière, afin d'évaluer l'impact des actions entreprises et de maintenir un niveau de sensibilisation des différents acteurs. Une stratégie globale intégrant organisation, éducation et restriction des prescriptions, pourrait permettre une réelle amélioration des taux de conformité des pratiques d'antibioprophylaxie.

### Etat des connaissances actuelles sur le sujet

- L'antibioprophylaxie (ATBP) est l'une des mesures spécifiques de prévention des infections du site opératoire dont l'impact est certain, mais le respect des recommandations reste faible, d'où l'intérêt de réaliser des audits clinique afin de promouvoir la prévention.

### Contribution de notre étude à la connaissance

- Notre étude est le premier audit clinique qui a permis de déterminer la conformité des pratiques de l'antibioprphylaxie chirurgicale dans notre hôpital;
- Ce travail constitue une opportunité pour les professionnels pour se familiariser avec les outils de l'évaluation des pratiques professionnelles;
- A l'issu de cette étude un plan d'amélioration de la qualité de l'antibioprphylaxie a été mis en place.
